# Successful Brain Delivery of Andrographolide Loaded in Human Albumin Nanoparticles to TgCRND8 Mice, an Alzheimer’s Disease Mouse Model

**DOI:** 10.3389/fphar.2019.00910

**Published:** 2019-08-22

**Authors:** Anna Rita Bilia, Pamela Nardiello, Vieri Piazzini, Manuela Leri, Maria Camilla Bergonzi, Monica Bucciantini, Fiorella Casamenti

**Affiliations:** ^1^Dipartimento di Chimica “Ugo Schiff,” University of Florence, Florence, Italy; ^2^Dipartimento di Neuroscienze, Psicologia, Area del Farmaco e Salute del Bambino (NEUROFARBA), University of Florence, Florence, Italy; ^3^Dipartimento di Scienze Biomediche, Sperimentali e Cliniche “Mario Serio,” University of Florence, Florence, Italy

**Keywords:** andrographolide, blood-brain barrier, human albumin nanoparticles, TgCRND8 mice, step-down inhibitory avoidance test, object recognition test, amyloid

## Abstract

Andrographolide (AG) was encapsulated in human albumin nanoparticles (AG NPs), and their crossing properties of the blood-brain barrier (BBB), brain distribution, and effects in TgCRND8 mice were evaluated. The development of appropriate NP formulations is mandatory because of the scarce BBB permeability properties of AG. Developed NPs had proper size (mean size: 159.2 ± 4.5 nm), size distribution (PDI nearby 0.12 ± 0.01), and ζ potential (-24.8 ± 1.2 mV), which were not affected by sodium fluorescein (NAF) loading. When AG was loaded to NPs, it slightly affected their size (210.4 ± 3.2 nm) and ζ potential (-20.3 ± 1.5) but not the PDI. Both NAF and AG had a remarkable encapsulation efficiency (more than 99%). The *in vitro* release of AG from the NPs reached the highest percentage (48%) after 24 h, and the Higuchi’s equation was found to be the best fitting model (R^2^ = 0.9635). Both AG and AG NPs did not alter the viability of N2a murine neuroblastoma cells when compared with the untreated control cells. In the step-down inhibitory avoidance test, AG NPs administered to TgCRND8 mice significantly improved their performance (P < 0.0001), reaching levels comparable to those displayed by wild-type mice. In the object recognition test, treated and untreated animals showed no deficiencies in exploratory activity, directional movement toward objects, and locomotor activity. No cognitive impairments (discrimination score) were detected in TgCRND8 mice (P < 0.0001) treated with AG NPs. After acute intravenous administration (200 µl), NPs loaded with the probe NAF were detected in the brain parenchyma of TgCRND8 mice. Immunofluorescent analyses evidenced the presence of NPs both in the pE3-Aβ plaque surroundings and inside the pE3-Aβ plaque, indicative of the ability of these NPs to cross the BBB and to penetrate in both undamaged and damaged brain tissues. Furthermore, the immunohistochemical analysis of GFAP-positive astrocytes in the hippocampus of Tg mice evidenced the anti-inflammatory activity of AG when AG NPs were intraperitoneally administered. AG was not effective in counteracting amyloid Aβ aggregation and the resulting toxicity but significantly decreased the oxidative stress levels. In conclusion, AG NPs have extraordinary versatility, nontoxicity, nonimmunogenicity, strong biocompatibility, high biodegradability, and astonishing loading capacity of drug.

## Introduction

Dementia, an umbrella term to describe symptoms of impairment in memory, communication, and thinking, has been declared by the World Health Organization (WHO) as a priority condition through the Mental Health Gap Action Programme. The cost of dementia according to the WHO, treating and caring for people with dementia, currently costs the world more than US$ 604 billion per year, but prevalence and incidence projections indicate that the number of people with dementia will continue to grow. An estimate of the future cost of dementia in Europe is a rise of 43% from 2008, reaching 250 billion Euros in 2030 (World Health Organization, 2012). The WHO’s report has been recently confirmed by the Alzheimer’s Disease International (ADI) research, which has estimated nearly double number of patients every 20 years, up to 65.7 million in 2030 and up to 115.4 million in 2050. Much of the increase will be in developing countries, the fastest growth in the elderly population is taking place in China, India, and their south Asian and western Pacific neighbors. The most common cause of dementia among elderly adults is Alzheimer’s disease (AD), currently affecting in developed countries approximately 30 million aged people and is expected to increase dramatically by 2050—when people aged 60 and over will account for 22% of the world’s population with four-fifths living in Asia, Latin America, or Africa (Prince et al., 2015).

Main clinical symptoms of AD include memory impairment and global cognitive deficits that can lead to dementia with the disease progression and noncognitive symptoms, especially loss of motor functions, such as gait disturbances, disturbed activity level and balance. Although the exact etiology of AD is not fully understood, substantial evidence indicates that amyloid-β peptide (Aβ), derived from sequential cleavage of amyloid protein precursor (APP) by β- and γ-secretases, plays a central role in the pathogenesis of AD (Hardy and Higgins, 1992). To date, there is no effective treatment for AD, and current therapeutic strategies only alleviate or treat disease symptoms but do not treat the underlying disease or delay its progression. Accordingly, researchers are looking for new multitarget drugs and combinatorial therapies to treat AD, including anti-inflammatory, antioxidant, and anti-amyloid approaches. Hence, studies are carried out using several cell lines associated with inherited APP mutations, neurotoxicity of soluble oligomeric Aβ when applied to neurons, and APP-overexpressing mice that recapitulate certain neuropathologic and behavioral features of AD.

**Figure 1:**
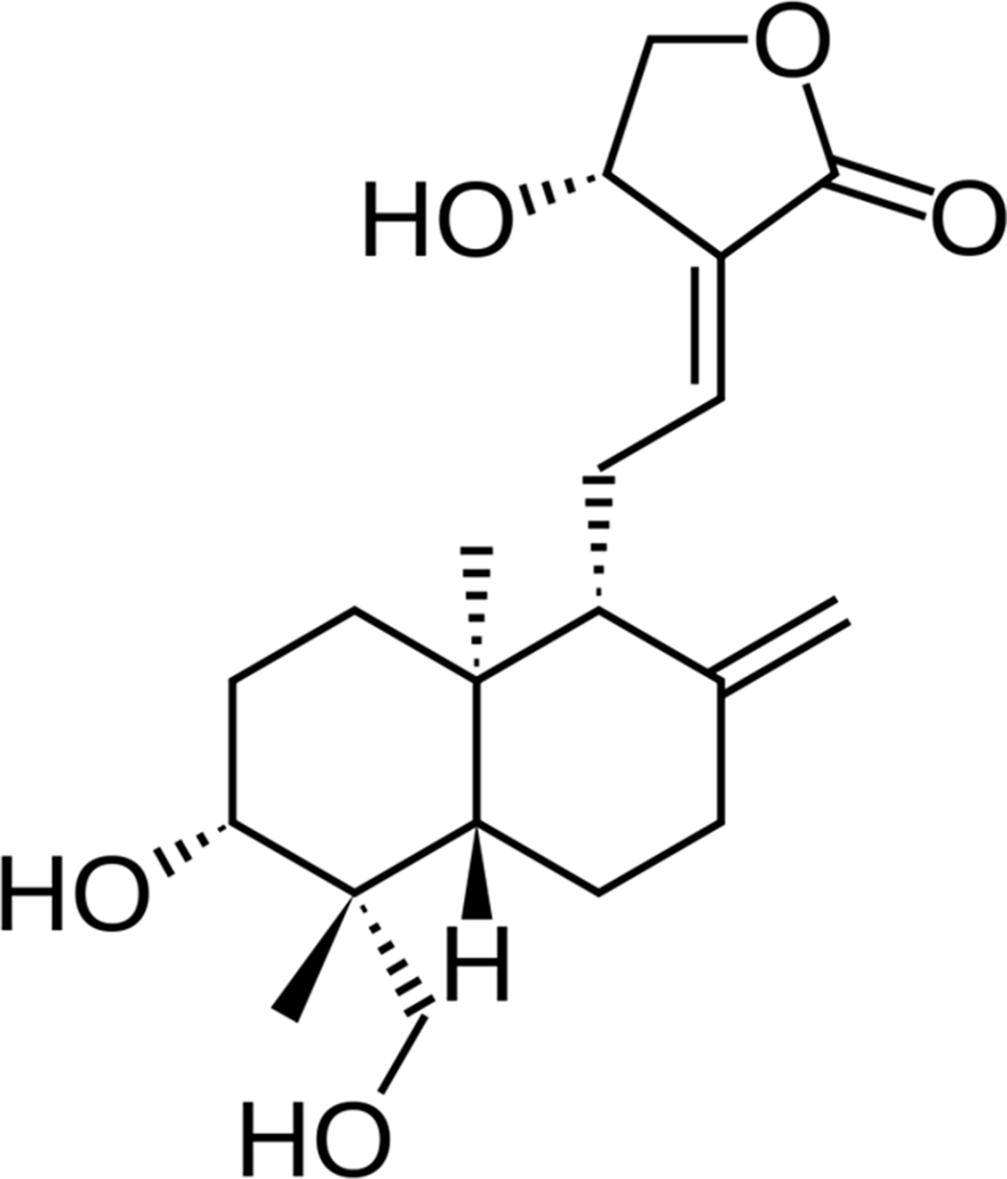
Structure of andrographolide (AG).

In this study, a natural product, andrographolide (AG, **Figure 1**), the main labdane diterpenoid from the Asian medicinal plant *Andrographis paniculata*, was investigated for its potential role in the treatment of AD patients. AG use looks promising for the broad range of multifunctional properties, principally antioxidant, neuroprotective, and anti-inflammatory (Asprea et al., 2019). Hence, inhibition of lipopolysaccharide-induced overexpression of HMGB1, TLR4, NFκB, COX-2, and iNOS and release of inflammatory mediators in primary mix glial culture adult mice prefrontal cortex and hippocampus region are reported for AG. Active microglial and reactive astrocytic markers can be also downregulated after AG treatment. In addition, AG decreases the expression of proapoptotic genes and enhances that of antiapoptotic genes, reducing neurodegeneration (Das et al., 2017; Kishore et al. 2017). AG has a neuroprotective role in a rat model of permanent cerebral ischemia (Chan et al., 2010) and prevents neuropathological changes in the APPswe/PS1ΔE9 mouse model, improving the spatial memory (Serrano et al., 2014). AG also induces the proliferation and the generation of new neurons in the adult hippocampus of wild-type (wt) and APPswe/PS1ΔE9 mice (Varela-Nallar et al., 2015).

Besides these recent studies highlighting the numerous different molecular targets of AG and their possible implications for Alzheimer’s disease treatment, AG has a low bioavailability that can greatly limits its biodistribution and localization and a very short biological half-life (t½ = 2 h) (Asprea et al., 2019). Recently, we have reported AG scarce permeability across the human monoculture *in vitro* blood-brain barrier (BBB) model based on human immortalized hCMEC/D3 cell line (Guccione et al., 2017). The experimental data confirmed that free AG did not affect cell layer integrity but did not significantly permeate the human immortalized hCMEC/D3 cell line (Guccione et al., 2017). This behavior was also confirmed by *in silico* predictions mainly because of the presence of five oxygen atoms in AG structure (ideally the number of oxygen atoms should not exceed three for ‘‘BBB + drugs”), with a rather high molecular polar surface area (above the threshold value of 70 Å, which clearly disfavors BBB permeation). The above data suggested that AG could represent a proper candidate to develop smart delivery systems for targeting the brain (Bilia et al., 2017, Bilia et al., 2018). We focused on nanoscale systems, human albumin polymeric nanoparticles (NPs), as a potential successful strategy able to increase stability and biopharmaceutical characteristics of AG, enhancing its penetration across the BBB because of their adsorptive transcytosis, targeting specific brain sites, and increasing therapeutic efficacy.

Accordingly, NPs loaded with AG (AG NPs) were administered to TgCRND8 mice, which express a transgene incorporating both the Indiana mutation (V717F) and the Swedish mutation (K670N/M671L) in the human amyloid-β protein precursor (APP) gene. The TgCRND8 mouse is a well-known model for reproducing important features of AD, including amyloid plaques and cognitive deficits (Bellucci et al., 2006). Interestingly, these mice display profound cognitive deficits at 2–3 months of age when few AD Aβ neuropathologies can be observed (Chishti et al., 2001).

Furthermore, to better understand the biological effects of AG, the potential ability of AG to modulate Aβ aggregation and to destabilize amyloid fibrils blocking the neurotoxicity of Aβ peptide, inhibiting a key step in the pathogenesis of AD, was investigated. Indeed, because of the recognized importance of misfolded aggregates in amyloid diseases, in recent years, the antiaggregation and protective effects of many different natural substances have been investigated, although with very variable results. Hence, numerous compounds have been shown to be active in the test tube, but their effectiveness *in vivo*, particularly in the real disease in humans, results were limited because of their poor stability and bioavailability.

## Materials and Methods

### Chemicals and Reagents

Human serum albumin, AG (purity 98%), NAF, sodium chloride (NaCl), sodium hydroxide (NaOH), phosphate buffered saline (PBS) bioperformance certified, dimethyl sulfoxide (DMSO), acetonitrile HPLC grade, methanol HPLC grade, formic acid analytical grade, hydrochloric acid (HCl) analytical grade, and ethanol analytical grade were purchased from Sigma-Aldrich (Milan, Italy). Water was purified by a Milli-Q Plus system from Millipore (Milford, CT, USA). Phosphotungstic acid (PTA) was from Electron Microscopy Sciences (Hatfield, PA, USA). Dialysis kit was from Spectrum Laboratories, Inc. (Breda, The Netherlands). RHB (150 mM NaCl, 2.2 mM CaCl2, 0.2 mM MgCl2, 5.2 mM KCl, 2.8 mM glucose, 5 mM HEPES, 6 mM NaHCO3) was prepared in-house, adjusted to pH 7.4, filtered, and stored at 4°C.

### Production and Characterization of NPs

#### Preparation of NAF NPs

Fluorescent human albumin NPs (NAF NPs) were prepared by thermal cross-linking (Bergonzi et al., 2016). Briefly, ethanol (4 ml) was added at a rate of 1 ml/min to 10 mM NaCl solution (4 ml) of human albumin (0.5% w/v) and NAF (0.5% w/v) under magnetic stirring (500 rpm) at room temperature. Stirring was continued at 90°C for 1 h to induce the thermal cross-linking of albumin. The obtained fluorescent NPs were purified by three cycles of centrifugation (1,358 g, 30 min) and dispersed in NaCl 10 mM. The concentration was set to 1 mg NAF/mL suspension. The suspension was used as such after quantification of NAF.

#### Preparation of AG NPs

AG NPs were obtained by thermal cross-linking, optimizing the method recently reported in our previous work (Guccione et al., 2017). In brief, 20 mg of AG were solubilized in 4 ml of ethanol, while human albumin (0.5% w/v) was dissolved in 4 ml of 10 mM NaCl. The AG solution was added at a rate of 1 ml/min to human albumin solution under magnetic stirring (500 rpm). Stirring was prolonged for 1 h at 90°C to obtain NPs. The resulting suspension was purified by centrifugation (1,358 g, 30 min × three times). The centrifuged particles were dispersed in NaCl 10 mM to obtain a final concentration of 1 mg AG/ml of suspension. The suspension was used as such after quantification of AG.

#### Yield of NPs

The yields of each NP preparation process were determined as the weight percentage of the final product after drying compared with the total amount of materials used for the formulation.

#### HPLC-DAD and HPLC-FLD Methods

The quantification of AG was performed on an HP 1200 liquid chromatograph equipped with a DAD detector. The column was a 150 mm × 4.6 mm i.d., 5 µm Kinetex EVO, RP18. The mobile phases were (A) CH_3_CN and (B) formic acid/water pH 3.2. Flow rate was 0.8 ml/min, and temperature was set to 27°C. The following gradient profile was utilized: 0–2 min, 5–15% A, 95–85% B; 2–5 min, 15% A, 85% B; 5–7 min 15–50% A, 85–50% B; 7–12 min, 50% A, 50% B; 12–15 min, 50–30% A, 50–70% B; 15–20 min, 30% A, 70% B; 20–25 min, 30–5% A, 70–95% B with equilibration time of 5 min. The UV/vis spectra were recorded in the range 200–800 nm, and the chromatograms were acquired at 223 nm.

NAF (λex = 460 nm, λem = 515 nm, green) analyses were performed using an HPLC 1200 liquid chromatograph equipped with an FLD detector, with the column used for AG maintained at 27°C.

The mobile phases were (A) CH_3_CN and (B) formic acid/water pH 3.2. Flow rate was 0.8 ml/min. The following gradient profile was utilized: 0–3 min, 20% A, 80% B; 3–23 min, 20–80% A, 80–20% B; 23–25 min 80–100% A, 20–0% B; 25–27 min, 100–20% A, 0–80% B with equilibration time of 5 min.

The linearity range of responses of AG and NAF dissolved in CH_3_OH and water, respectively, was determined on five concentration levels using three injections for each level (AG from 0.05 to 20 µg/ml, NaF from 0.05 to 35 µg/ml). Both curves showed a coefficient of linear concentration above than 0.999. limit of quantitation (LOQ) and limit of detection (LOD) of AG resulted in 5.3 ng and 2.6 ng, respectively.

#### Encapsulation Efficiency and Loading Capacity

The amount of AG entrapped into NPs was calculated using an indirect method, previously described in our studies (Bergonzi et al., 2016; Grossi et al., 2016).

NPs were purified by ultracentrifugation for 75 min at 18,000 rpm and 4°C. Encapsulation efficiency (EE%) and the loading capacity (LC%) were calculated as follows:

EE% = (Total Drug-Free Drug)/(Total Drug) · 100
LC% = (Total Drug-Free Drug)/(Weight of NPs) · 100

AG quantifications were carried out on a reverse-phase HPLC system, as described in the previous session.

#### NPs Morphology, Shape, Size, and **ζ** Potential

NPs were characterized by transmission electron microscopy (TEM), dynamic light scattering (DLS), and electrophoretic light scattering (ELS). All dispersions of NPs were diluted 10 times before to be applied to a carbon film-covered copper grid. PTA solution (1% w/v) was used as contrast color. The samples were dried and then examined under a TEM JEOL 1010 electron microscope and photographed at an accelerating voltage of 64 kV.

DLS and ELS analyses were performed with a Zetasizer Nano series ZS90 (Malvern Instruments, Malvern, UK) equipped with a JDS Uniphase 22mW He-Ne laser operating at 632.8 nm, an optical fiber-based detector, a digital LV/LSE-5003 correlator, and a temperature controller (Julabo water bath) set at 25°C.

The hydrodynamic diameter of the particles (Zh) and the particle size distribution (polydispersity index, PDI) were analyzed using the ALV-60X0 software V.3.X provided by Malvern. The ζ potential, indicating the electric charge on the NPs surface and the physical stability of the formulations, was calculated by the Helmholtz-Smoluchowsky equation using the same instrument.

#### Storage Stability

Stability of AG NPs was investigated by monitoring their physical characteristics, namely hydrodynamic diameter, PDI, ζ potential, and EE% over 30 days at 4°C.

#### *In Vitro* Release Studies

*In vitro* release of AG was investigated employing the permeable membrane bag method using regenerated cellulose membranes with a molecular cutoff of 3.5 kD. Phosphate buffer solution (PBS, pH 7.4) was selected as release medium. Two milliliters of AGNPs was added into the membrane bag and placed in 200 ml of release medium. The system was incubated at 37°C with stirring at 150 rpm. At predetermined time intervals (0, 0.5, 1, 2, 3, 4, 6, 9, 24 h), 1 ml of the medium was withdrawn and replaced with the same volume of fresh release medium maintained at 37°C to preserve sink conditions. Released AG at each time interval was quantified using HPLC-DAD. The experiments were performed in triplicate.

AG release data were fitted using Korsmeyer-Peppas, Hixson Crowell, Higuchi, first-order and zero-order mathematical models to understand the release pattern of entrapped compound from developed NPs. The regression analysis was performed to obtain the best fit correlation.

In addition, NAF release was studied using the same method to evaluate the stability of fluorescent NAF NPs. The test time was set up to 3 h, according to the *in vivo* tests. At predetermined time intervals (0, 0.5, 1, 2, 3 h), 1 ml of the medium was withdrawn and replaced with fresh PBS. The samples were analyzed by HPLC-FLD.

### *In Vitro* Studies

#### Thioflavin T Assay

Aβ_1-42_ aggregates grown for different times (0, 3, 24, 48, and 72 h) in the absence or in the presence of AG were diluted with 20 mM phosphate buffer, pH 7.4, at 25°C to a 15-μM peptide concentration and supplemented with a small volume of a 1.0-mM Thioflavin T (ThT) solution to a 20 μM final ThT concentration. Then, each sample was transferred into multiple wells of a 96-well half-area, low-binding, clear bottom (200 μl/well), and ThT fluorescence was read at the maximum intensity of fluorescence of 485 nm using a Biotek Synergy 1H plate reader; the ThT fluorescence of buffer was subtracted from all samples’ fluorescence values. To ascertain any AG interference on ThT fluorescence, we performed control experiments where AG was added in pre-made Aβ42 aggregates either immediately before or after the addition of ThT solution.

#### TEM Imaging

Five-microliter aliquots of aggregating Aβ42 in the presence or in the absence of AG were withdrawn at different aggregation times, loaded onto formvar/carbon-coated 400 mesh nickel grids (Agar Scientific, Stansted, UK) and negatively stained with 2.0% (w/v) uranyl acetate (Sigma-Aldrich). The grid was air-dried and examined using a JEM 1010 transmission electron microscope (TEM) at 80 kV excitation voltage.

#### MTT Assay

N2a murine neuroblastoma cells (ECACC) were seeded into 96-well plates at a density of 5,000 cells/well in MEM supplemented with non-essential amino acids, 10% FCS, antibiotics, and glutamine, cultured for 24 h. To access the cell viability after AG and AG NP exposure, an MTT assay was performed. The cells were treated with different concentrations of either AG or AG NPs (1, 2, and 3 µM) for increasing time periods (4, 24, and 48 h) and evaluated for viability by the MTT assay, as previously described (Grossi et al., 2013). After 24 h of incubation, the culture medium was removed, and the cells were incubated for 1.0 h at 37°C in 100 μl of serum-free DMEM without phenol red, containing 0.5 mg/ml MTT. Then, 100 μl of cell lysis solution (20% SDS, 50% N,N-dimethylformamide) was added to each well, and the samples were incubated at 37°C for 2 h to allow complete cell lysis. The absorbance of the blue formazan resulting from MTT reduction was read at 570 nm using a spectrophotometric microplate reader. The final absorption values were calculated by averaging each sample in triplicate and subtracting the blank (100 μl of MTT solution + 100 μl of lysis solution) value.

#### Immunofluorescence

Subconfluent N2a cells grown on glass coverslips were treated for 24 h with Aβ42 samples (2.5 μM) grown in the presence or in the absence of AG and then washed with PBS. GM1 labeling was performed by incubating the cells with 10 ng/ml CTX-B Alexa 488 in complete medium for 10 min at room temperature. Then the cells were fixed in 2.0% buffered paraformaldehyde for 10 min and permeabilized by treatment with a 1:1 acetone/ethanol solution for 4.0 min at room temperature, washed with PBS, and blocked with PBS containing 0.5% BSA and 0.2% gelatin. After incubation for 1.0 h at room temperature with rabbit anti-Aβ42 polyclonal antibody diluted 1:600 in blocking solution, the cells were washed with PBS for 30 min under stirring and then incubated with Alexa 568-conjugated anti-rabbit secondary antibody (Molecular Probes) diluted 1:100 in PBS. Finally, the cells were washed twice in PBS and once in distilled water to remove non-specifically bound antibodies. Cell fluorescence was imaged using a confocal Leica TCS SP5 scanning microscope (Leica, Mannheim, Ge) equipped with a HeNe/Ar laser source for fluorescence measurements. The observations were performed using a Leica Plan 7 Apo X63 oil immersion objective (Nosi et al., 2012).

### *In Vivo* Studies

#### Ethics Statement

Transgenic (Tg) CRND8 mice harboring a double-mutant gene of APP695 (Chishti et al., 2001) and wt control littermates were used in accordance with the principles of the Basel Declaration and the Working document on genetically altered animals—CORRIGENDUM of 24 January 2013—of the National Competent Authorities for the implementation of Directive 2010/63/EU on the protection of animals used for scientific purposes. According to the Italian Regulation (D.Lvo 26/2014), the protocol was revised and approved by the Animal Welfare Body of the University of Florence and licensed by the Italian Competent Authority (Italian Ministry of Health, license number 152/2014-B).

#### Animals

Four-month-old Tg and wt mice (n = 6/group/genotype, equally divided for sex) were used. Animals were housed in macrolon cages with food and water *ad libitum* and maintained on a 12-h light/dark cycle at 23°C. All efforts were made to minimize the number of animals used and their suffering.

#### Systemic Administration of NAF NPs and AG NPs

##### Intravenous Injection of NAF NPs

First, we checked for the ability of NPs to cross the BBB in TgCRND8 mice acutely dosed with NAF NPs (200 µl, 1 mg/ml solution) or vehicle (0.9% NaCl) by intravenous (i.v.) injection by the tail vein. Three hours after the administration, mice were perfused transcardially with saline solution (0.9%) to remove blood components and then with 4% ice-cold paraformaldehyde in 0.1M PBS, pH 7.2, under deep anesthesia [Zoletil 80/mg/kg, intraperitoneally (i.p).].

##### Intraperitoneal Administration of AG

Second, Tg and wt mice were administered i.p. with AG NPs dissolved in saline (4 mg/kg) for 4 weeks (three times a week). The animals were weighted once a day, the body weight was recorded, and the dose to be administered i.p. was recalculated according to the body weight. After completing the behavioral tests, Tg mice were perfused transcardially with saline solution (0.9%) to remove blood components and then with 4% ice-cold paraformaldehyde in 0.1 M PBS, pH 7.2, under deep anesthesia (Zoletil 80/mg/kg, i.p).

#### Behavioral Experiments

After AG NP administration, treated and untreated Tg and wt mice were behaviorally tested.

##### Step-Down Inhibitory Avoidance Test

The apparatus and procedures used for the step-down inhibitory avoidance test were previously described (Grossi et al., 2013). On day 1 (training test, TT), each mouse was placed on the platform and received an electric shock for 3 s when it stepped down placing all paws on the grid floor. Responsiveness to the punishment in the TT was assessed by animal vocalization; only those mice that vocalized touching the grid (about 70% of mice) were used for retention test (RT). Twenty-four hours after TT, each mouse was placed on the platform again (RT). The latencies were measured, considering 180 s as the upper cutoff, during TT and RT. The tests were carried out between 10:00 A.M. and 1:00 P.M.

##### Object Recognition Test

The apparatus and procedures used for object recognition test (ORT) were previously described (Grossi et al., 2013). Briefly, a session of two trials (T1 and T2) at 60-min interval was given on the test day. In T1, the time spent by each mouse exploring two identical 8.0 cm side gray cubes presented for 10 min in two opposite corners of the box was recorded. During T2, an 8.0 cm side gray cylinder replaced one of the cubes, and the mice were left in the box for 5 min. The times spent for the exploration of the familiar (F) and the new (N) object were recorded, and a discrimination score (D = N/N + F) was calculated.

#### Animal Tissue Processing

After completing the behavioral tests, the mice were transcardially perfused as reported above, brains were quickly extracted and fixed in phosphate-buffered (PBS) 4% paraformaldehyde (pH 7.4) for 24 h at 4°C. Subsequently, brains were rinsed with PBS for 24 h, dehydrated using an automated machine, and paraffin embedded. Coronal sections (5.0 µm) were cut using a microtome and mounted on slices.

#### Histochemistry and Fluorescent Immunohistochemistry

Histochemical and fluorescent immunohistochemical analyses were performed on 5.0-µm coronal paraffin-embedded sections, as previously described (Luccarini et al., 2015). Pyroglutamate3 (pE3)-amyloid-β (pE3-Aβ)-positive plaques were detected by the N3pE antibody (dilution 1:200; IBL International, Hamburg, Germany), and astrocytes were visualized by the GFAP antibody (1:500 dilution; Millipore, Billenca, MA, USA). The sections were coverslipped using ProLong (Lyfe Technologies, NY, USA) mounting medium with DAPI for nuclear staining. Representative images were acquired by an Olympus BX63 microscope coupled to CellSens Dimension Imaging Software version 1.6 (Olympus, Milan, Italy).

### Statistical Analysis

Two- and one-way ANOVA, plus Bonferroni’s *post hoc* test, were used to analyze step-down inhibitory avoidance and ORT data, respectively, and Student’s t test for MTT and ROS assays. Calculations were performed using either Excel or GraphPad Prism version 5.0 for Windows, with statistical significance established at *P* < 0.05. Data are reported as mean values ± standard error of the mean (SEM).

## Results

### Preparation and Characterization of NPs

**Figure 2:**
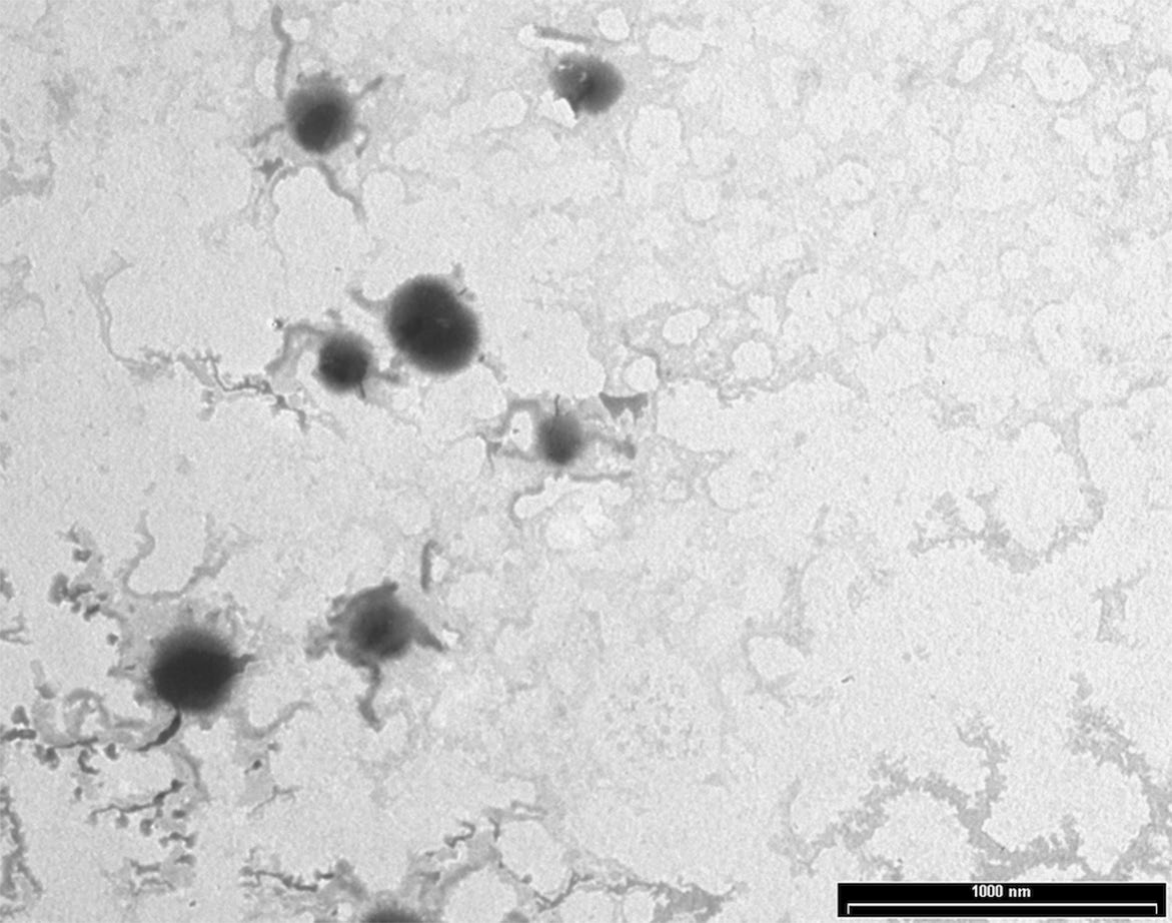
TEM analysis of AG NPs.

NPs were produced using a thermal cross-linking method at physiological pH, without the requirement of solvents or glass beads. Briefly, NPs were obtained by heating at 90°C for 1 h human serum albumin. AG NPs and NAF NPs were prepared by adding AG or NAF before the heating process. Stability of both NAF and AG was good during this process, giving very high yields of loading. The developed method of synthesis was simple, environment friendly, and with a high reproducibility in terms of yield and NPs size (n = 20). The yields of preparative process were ca. 80% for NAF NPs and ca. 87% for AG NPs. The resulting NPs were roughly spherical, with a reasonably regular structure, as evidenced by TEM analysis, which provided details on morphology (**Figure 2**). DLS analysis was consistent with TEM analysis in terms of size, confirming a monodispersed size distribution and, in addition, showed a negatively charged ζ potential for all NPs, even after AG or NAF loading. All the DLS data are reported in **Table 1**. AG and NAF loading and EE% were obtained by HPLC analysis using the analytical method described in the experimental part. Data are given in **Table 1**. It is remarkable that the mean size of unloaded NPs was ca. 150 nm, and it was only slightly affected by the NAF loading (mean size: 159.2 ± 4.5 nm). NAF NPs had a proper narrow size distribution (PDI nearby 0.12 ± 0.01), and physical stability was supposed by the high negative charge. AG NPs slightly affected the size of NPs (210.4 ± 3.2 nm) but not the PDI (0.10 ± 0.01), which was highly monodispersed. High physical stability was expected because of the suitable ζ potential values.

**Table 1:** Characterization of developed HA NPs in terms of mean diameter, polydispersity, ζ potential, yield of preparative process, encapsulation efficiency, and loading capacity (mean ± SD; n = 3).

Nanoparticles	Size (nm)	PDI	ζ Potential (mV)	EE%
NAF NPs	159.2 ± 4.5	0.12 ± 0.01	-24.8 ± 1.2	99.5 ± 0.1
AG NPs	210.4 ± 3.2	0.10 ± 0.01	-20.3 ± 1.5	99.1 ± 0.2

### Physical and Chemical Stability of AG NPs

Stability of the developed AG NPs was investigated in terms of dimensions, PDI, ζ potential, residual drug loading (%) over 30 days at 4°C using the NP suspension. From the above data, storage did not affect any investigated parameters as reported in **Table 2**.

**Table 2:** Stability of developed AG NPs in terms of mean diameter, polydispersity, ζ potential, and residual drug loading (%) (mean ± SD; n = 3).

Parameter	AG NPs			
	After 7 days	After 14 days	After 21 days	After 30 days
Size (nm)	210.8 ± 1.2	211.4 ± 2.5	212.6 ± 0.8	212.9 ± 2.3
PDI	0.10 ± 0.01	0.12 ± 0.02	0.09 ± 0.01	0.10 ± 0.01
ζ potential (mV)	-20.5 ± 4.4	-20.1 ± 2.7	-21.3 ± 4.8	-20.2 ± 1.1
Residual drug loading (%)	99.1 ± 0.2	98.8 ± 0.4	98.8 ± 0.3	98.7 ± 0.2

### *In Vitro* Release Studies From AG NPs

**Figure 3:**
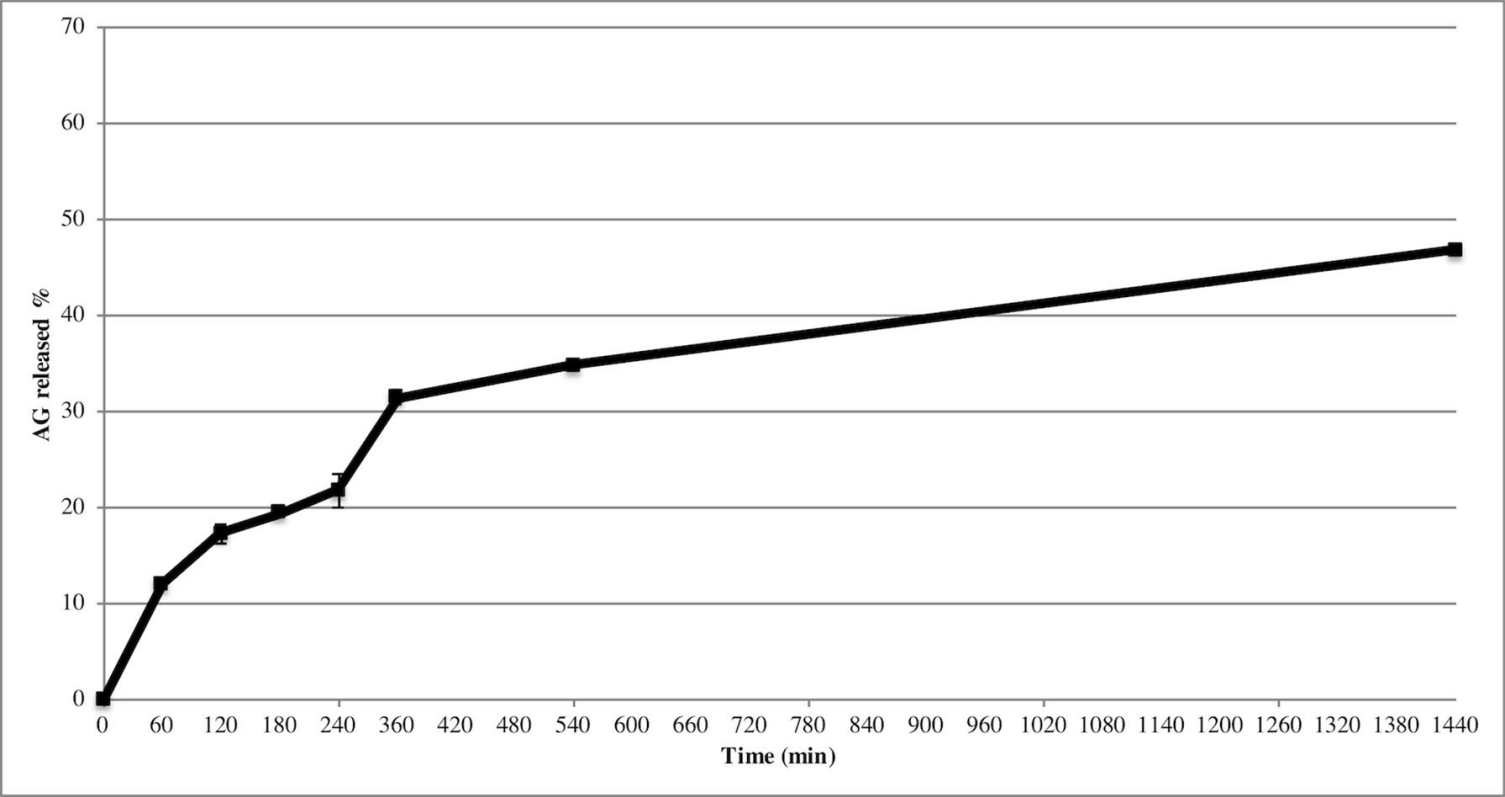
Release of AG from NPs in PBS, pH 7.4. Data displayed as mean ± SD (n = 3).

**Figure 4:**
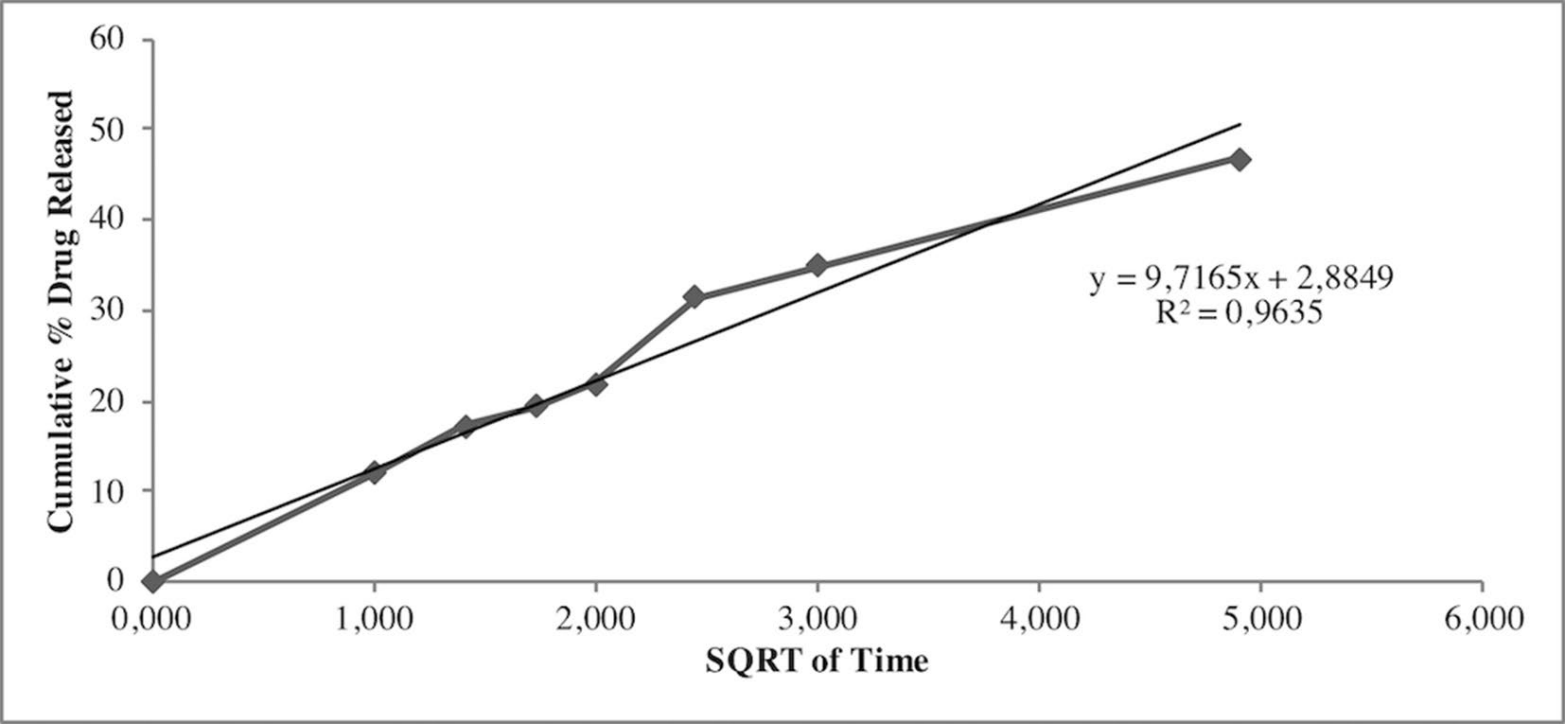
Higuchi mathematical equation model best fitted the AG release data from NPs.

Release study was developed to quantify the amount and extent of AG release from NPs. The test was carried out during 24 h using the membrane bag method in a PBS solution at pH 7.4 to mimic the sink conditions. **Figure 3** depicts the AG released during time from AG NPs. The curve was typical for controlled release without a burst effect at the beginning of the test, which was in agreement with a complete entrapment of AG into NPs. The highest percentage of released AG during 24 h test was ca. 48%. Calculation of the percentage of AG released in the medium used the formula previously reported (Guccione et al., 2017). Different mathematical equation models were investigated to better describe AG process of release from NPs and determine the kinetics. The release data were fitted to many kinetic models, and the Higuchi kinetic was shown to be the best fitting model, as reported in **Figure 4**.

### *In Vitro* NAF Release Studies From NAF NPs

NAF release was studied using the same method for AG to evaluate the stability of fluorescent NPs. The test time was set to 3 h, the same time of the *in vivo* tests. At predetermined time intervals (0, 0.5, 1, 2, 3 h), 1 ml of the medium was withdrawn and replaced with fresh PBS. The samples were analyzed using HPLC-FLD as reported in the experimental part. No traces of NAF were detected during the experimental time, evidencing the lack of NAF release from NPs.

### Effects of AG on Aβ Amyloid Aggregation

**Figure 5:**
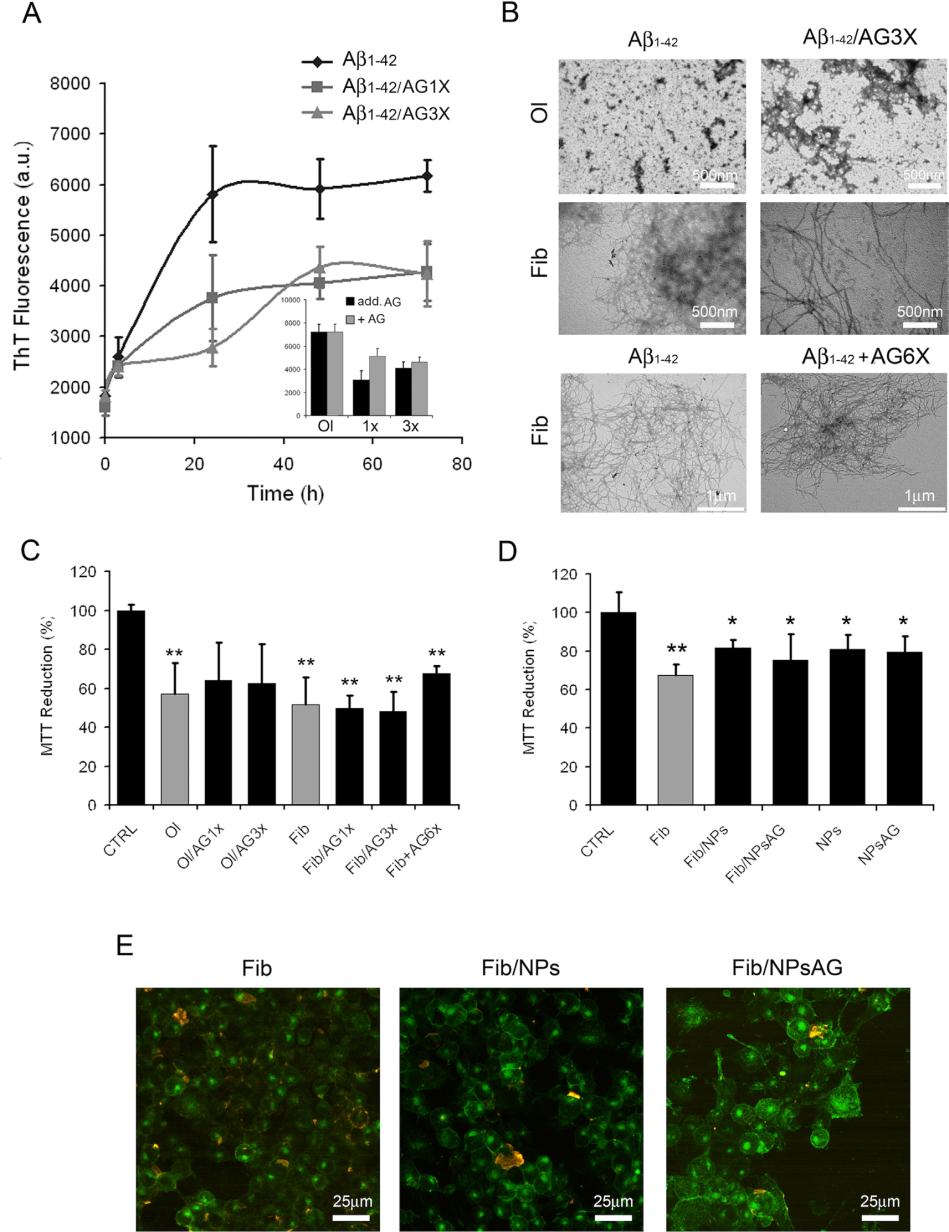
Effects of AG on Aβ_1-42_ aggregation process and resulting cytotoxicity. Aβ aggregation process followed by ThT fluorescence assay **(A)**. The interference of AG with the ThT fluorescence assay was evaluated by comparing the emission fluorescence spectra intensity of Aβ_1-42_ aggregates with respect to when AG was added together to the probe or immediately after the probe (A, inset). TEM pictures taken from Aβ_1-42_ aggregation mixture after 24 and 72 h of incubation in the absence or in the presence of 3X-AG **(B)**, first and second lines). TEM pictures taken from preformed Aβ fibrils 72 h aged and successively treated with 6X-AG. The scale bars are shown. Cell viability assessed by the MTT reduction assay **(C–D)**. N2a cells treated for 24 h with 2.5 µM Aβ solution obtained at 24 and 72 h of aggregation time. Each sample was obtained in the absence or in the presence of 1X- or 3X-AG or AG NPs. For the MTT assay, preformed fibrils treated with 6X-AG were also tested **(C)**. Error bars indicate the standard deviation of independent experiments carried out in triplicate. Statistical analysis was performed using Student’s t-test: *P < 0.05, **P < 0.01 versus untreated cells and *P < 0.05 versus treated cells with Aβ_1-42_ aggregates grown without AG. The Aβ fibrils grown both in the absence and in the presence of AG NPs or NPs bind to the plasma membrane. Z-projection of N2a cell images by Aβ_1-42_ immunostaining (red) and GM1 staining (green). The scale bars are shown **(E)**.

The data presently available in the literature suggest that AG could provide a protective and therapeutic effect against a number of pathologies, including AD. Such a protection could be caused, at least in part, by its possible ability to inhibit the amyloid aggregation process of Aβ peptide. For this aim, the kinetics of fibril formation were monitored by following the increase of ThT fluorescence emission intensity at 485 nm during the *in vitro* Aβ fibrillation process in the absence or in the presence of two different concentrations of AG (1X- and 3X-AG). **Figure 5A** shows that the presence of AG in Aβ aggregation solution led to a decrease in the apparent ThT fluorescence because of its quenching effect (**Figure 5A** inset). This evidence indicates that AG does not alter significantly the ThT fluorescence spectra from fibrillar Aβ alone. The absence *in vitro* of antiaggregation activity of AG was confirmed by TEM micrographs (**Figure 5B**). TEM images indicate that the protein after 24 h of aggregation either alone or aggregated in the presence of 3X-AG exists as globular micelle-like and prefibrillar assemblies, whereas after 72 h of aggregation, the samples are populated mostly of mature fibrils in both conditions. Moreover, AG is unable to dissolve preformed Aβ fibrils even at 6X-AG concentration after 24 h of treatment, as indicated in **Figure 5B**. The toxicity of the same protein samples was assessed on N2a cells by using the MTT reduction assay. The amyloid assemblies obtained in the presence of AG or AG NPs and the Aβ fibrils obtained with 1X-, 3X-, and preformed fibrils treated with 6XAG induced in N2a cells a similar toxicity as well as amyloid Aβ (**Figures 5C, D**). Cell surface staining with GM1 in N2a cells and immunofluorescent labeling of Aβ showed that amyloid species obtained in the absence or in the presence of AG or AG NPs were similarly able to bind to the cell membrane (**Figure 5E**). All together, these results indicate that *in vitro*, the AG molecule is not effective to counteract directly amyloid Aβ aggregation and the resulting cytotoxicity.

### Effect of AG or AG NPs Treatment on Viability of N2a Cells

**Figure 6:**
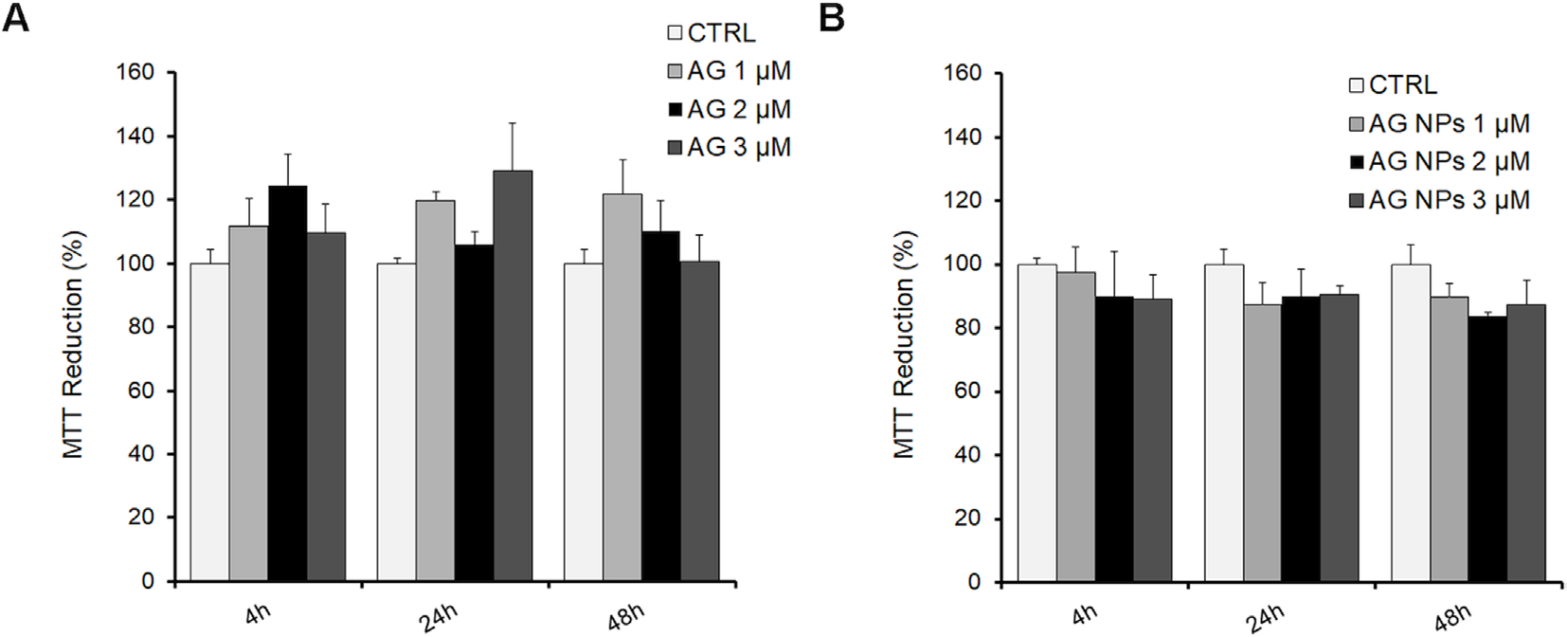
MTT assay in N2a cells exposed to AG **(A)** or AG NPs **(B)**. N2a cells were seeded into 96 well plates and exposed for 4, 24, or 48 h to 1.0, 2.0, or 3.0 µM of AG or AG NPs. Viability is expressed as a percentage with respect to that of control untreated cells. Data are reported as mean ± SEM.

N2a cells were exposed for 4, 24, or 48 h to 1, 2, and 3 µM AG (**Figure 6A**), AG NPs (**Figure 6B**), or vehicle to evaluate the toxicity of the treatment on cell viability by the MTT assay, which is based on its reduction by mitochondrial and membrane-associated dehydrogenases in metabolically active cells. No effect of all treatments on cell viability was found at the three time periods of AG or AG NP exposure as compared to that of control cells. The viability of untreated and vehicle-exposed N2a cells was not different, and thus the samples were pooled in the control value.

### *In Vivo* Experiments

**Figure 7:**
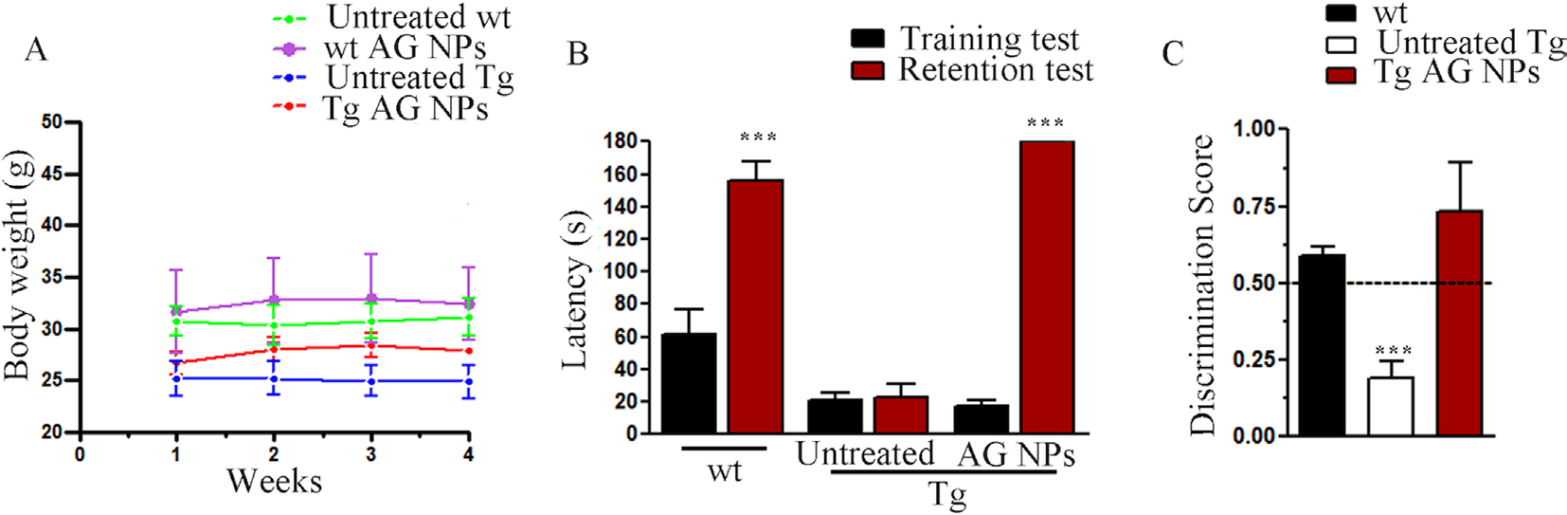
**(A)** Body weight: AG NPs treatment did not affect body weight of Tg and wt mice; **(B)** step-down inhibitory avoidance test, the training test (black bars) showed no significant differences between groups. The 24-h retention test (red bars) showed increased latencies in controls and AG NP-treated Tg mice, as compared to their respective training latencies and to the retention latencies of untreated Tg mice (***P < 0.0001). In untreated Tg mice, retention latencies were not significantly different from training latencies. **(C)** ORT test: in the T2 trial, the discrimination index of untreated Tg mice significantly differs from the discrimination index of all other groups (***P < 0.0001). The dotted line indicates the chance level performance. Number of animals: n = 6/group.

**Figure 8:**
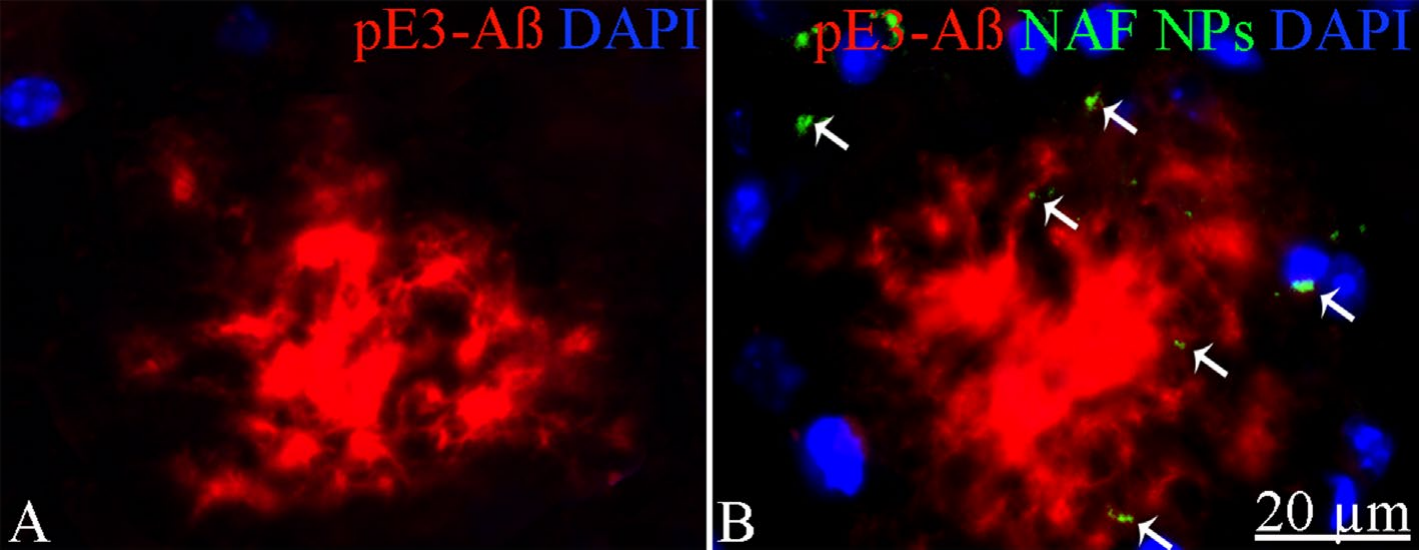
Immunohistochemical analysis of pE3-Aβ in the cortex of Tg mice. **(A)** Untreated; **(B)** treated with NAF NPs. Note the presence of NAF NPs (arrows) in the pE3-Aβ plaque surroundings and inside the pE3-Aβ plaque. Scale bar = 20 µm applies to both images.

**Figure 9:**
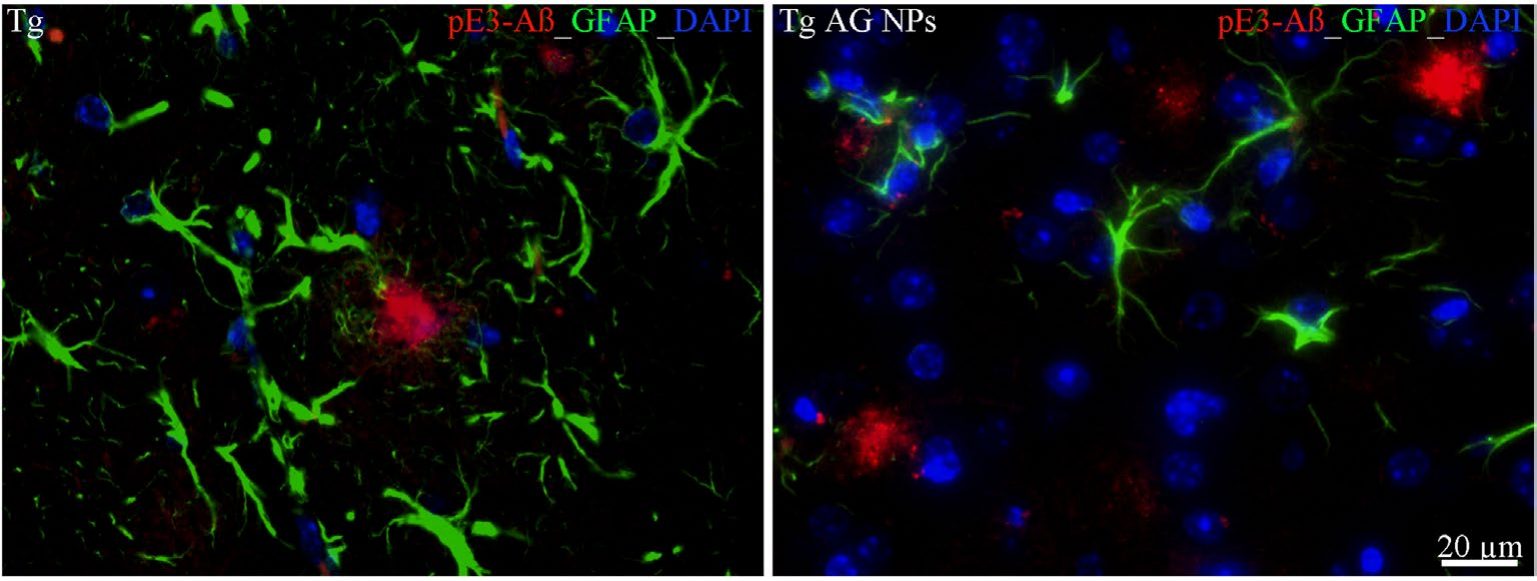
Immunohistochemical analysis of GFAP positive astrocytes in the hippocampus of Tg mice. Left panel: untreated Tg mice; right panel): treated (AG NPs). GFAP staining (green) revealed less reactive astrocytes, characterized by enlarged cell body, in the pE3-Aβ plaque (red) surroundings of treated (AG NPs) mice compared to untreated Tg mice. DAPI is in blue. Scale bar = 20 µm applies to both images.

To exclude potential AG NPs (4 mg/kg, administered i.p. for 4 weeks) toxicity, the body weight of the animals was recorded during the period of treatment. We found that the AG NPs were well tolerated, and no evident side effects were revealed, as shown by the body weight trend graph of the AG NPs-treated mice relative to that of the control represented by the vehicle (**Figure 7A**), and no animals died. To evaluate potential effects of AG NPs on cognitive functions and locomotor-exploratory abilities at the end of treatment, the animals were tested for behavioral performance in the step-down and ORT. In the step-down inhibitory avoidance test (**Figure 7B**), two-way ANOVA statistical analysis with genotype/treatment and retention time as the two variables revealed that there was a significant main effect for treatment/genotype [F(2,58) = 121.5, P < 0.001] and retention time [F(1,58) = 446.7, P < 0.001] and a significant interaction genotype/treatment × retention time [F(2,58) = 100.4, P < 0.001]. Latencies observed for untreated Tg mice in the step-down RT were significantly reduced when compared to those of wt mice (***P < 0.0001). AG NPs treatment of Tg mice significantly improved their performance (***P < 0.0001 vs. untreated Tg mice) to levels comparable to those displayed by wt mice. In the ORT, treated and untreated animals showed no deficiencies in exploratory activity, directional movement toward objects, and locomotor activity and no cognitive impairments (discrimination score) were detected in AG NPs-treated Tg mice (***P < 0.0001 vs. untreated Tg mice) (**Figure 7C**). Next, to evaluate the potential therapeutic use of AG NPs in neurodegenerative diseases, such as AD, we analyzed the ability of these NPs to penetrate the BBB and to migrate in the brain parenchyma in the TgCRND8 mouse model of Aβ deposition. Thus, the penetration into the brain of NAF NPs (green), 200 µl administered acutely i.v., was investigated. Three hours after the administration of the fluorescent NPs (**Figures 8A, B**), the formulation was detected in the brain parenchyma (arrows) of Tg mice. Interestingly, in immunofluorescent analyses with the N3pE antibody that recognizes pE3-Aβ plaque, NAF NPs (arrows) were detected both in the pE3-Aβ plaque (red) surroundings and inside the pE3-Aβ plaque (**Figure 8B**). This is indicative of the ability of NPs to cross the BBB and to penetrate undamaged and damaged brain tissues. Moreover, we found, in immunohistochemical study with the GFAP antibody (green), that the AG NPs-induced amelioration of cognitive functions was associated with a reduced astrocyte reaction, revealing fewer reactive astrocytes with enlarged cell body in the pE3-Aβ plaque (red) surroundings and brain parenchyma in the hippocampus of AG NPs-administered Tg mice compared to untreated Tg mice (**Figure 9**). Overall, these data provide strong evidence for the AG NPs efficacy on cognitive functions and further support the anti-inflammatory activity of AG. AG NPs might represent valuable and safe vectors for brain delivery systems, and our present findings indicate that AG can be transported across the BBB by NPs and released within the brain in a therapeutically relevant concentration.

## Discussion

Neurodegenerative diseases, including AD, are the largest growing area of neurological research still in search of an effective therapy to suppress the main clinical signs and/or to delay the occurrence of severe pathologies. AD, the most common form of dementia affecting many aged people, is characterized by progressive memory loss and neuropathological changes in specific regions of the brain (Selkoe, 2013). Recent studies have proposed natural products to treat and prevent the progression of the disease (Feart et al., 2013; Ng et al., 2015). These are principally represented by plant polyphenols (Stefani and Rigacci, 2013), which interfere with the amyloid aggregation of Aβ peptides (Serrano et al., 2014; Casamenti and Stefani, 2017), whose mechanism is mainly redirecting fibrillar aggregation toward the formation of amorphous harmless aggregates without the appearance of toxic oligomeric entities (Ladiwala et al., 2011). Docking simulations are consistent with the hypothesis of the π-π stacking between aromatic phenyl rings of polyphenols and aromatic residues of the proteins (Gazit, 2002; Porat et al., 2006). Recent studies demonstrated that curcumin intercalates in a planar-lengthwise fashion parallel to the fibril axis and disrupts interstrand beta sheet chain interactions, precluding ordered fibril assembly (Rao et al., 2015). Other studies suggested the presence of o-quinone-mediated covalent adduct formation from certain flavonoid or catechol-containing compounds with amyloid proteins (Velander et al., 2017). Additional covalent mechanisms have also been reported between nucleophilic amines and thiols of amyloidogenic proteins and electrophilic reactive groups on inhibitors such as aldehydes (Li et al., 2009; George et al., 2013). Overall, the presence of aromatic/hydrophobic moieties and hydrogen-bonding elements along flat extended structures commonly observed on aggregation inhibitors appears to be determinant shared structural features for inhibition activity.

AG, a bicyclic labdane diterpene from the Chinese medicinal plant *Andrographis paniculata*, has been recognized for neuroprotective effects against inflammation-mediated neurodegeneration (Wang et al., 2004; Suebsasana et al., 2009), oxidative stress in the brain (Das et al., 2009), and cerebral ischemia (Chan et al., 2010), but it suffers from low BBB permeation, as recently reported (Guccione et al., 2017). Furthermore, Serrano et al. (2014) reported that, in young AβPPswe/PS-1 mouse model of AD, AG affects amyloid plaque maturation in hippocampi and cortices, whereas in old animals, AG was not able to decrease the amyloid deposits and Aβ oligomer levels. Moreover, Serrano and coworkers showed that AG induces a reduction of tau phosphorylation around the Aβ oligomeric species and recovers spatial memory functions likely by inhibition of GSK-3 activity. In addition, it is reported in the literature that AG reduces several neuropathological markers of AD in *Octodon degus*, the only wt South American rodent to develop Alzheimer’s-like pathology in older age (Rivera et al., 2016).

To the best of our knowledge, further studies are needed to elucidate the biochemical and pharmacological mechanisms that underlie the observed AG activities, understanding if the molecule is able to prevent or modulate directly the amyloid aggregation process. Indeed, this molecule could recover the cognitive performances and reduce the neuropathological hallmarks of AD by attenuating the toxicity of misfolded Aβ, by converting the toxic assemblies into harmless species, or affect the secondary cellular signaling pathways triggered by the accumulation of misfolded species, for instance, inflammation and oxidative stress.

In the present study, AG NPs suitable for *in vivo* systemic administration were developed, and NAF NPs were evaluated for their crossing properties of the BBB and brain distribution in TgCRND8 mice. AG NPs were suitable for parenteral administration, in terms of shape, size, surface charge, and AG loading capacity. The settled method of synthesis through heat was environment friendly, simple, highly reproducible, appropriate in terms of yield, and easy to scale up. NPs were also very stable in terms of physical and chemical properties. Storage for 30 days of AG NPs suspension did not affect any physical or chemical parameters.

Release studies were also performed to investigate AG diffusion performance in the developed NPs and its release in the physiological medium. Results indicated no burst effect, in agreement with the absence of superficial adsorbed AG. By contrast, NPs were able to release AG for a prolonged period. The quantitative analysis of released AG was investigated using several mathematical equation models, including first-order and zero-order mathematical models, Korsmeyer-Peppas, Higuchi, Hixson Crowell, to understand the release pattern of entrapped compound from developed NPs. The regression analysis gave the best fit with the Higuchi’s equation. In particular, the Higuchi model is based on different hypotheses that i) initial drug concentration in the matrix is much higher than drug solubility, ii) drug diffusion takes place only in one dimension (edge effect should be avoided), iii) drug particles are much smaller than thickness of system, iv) swelling of matrix and dissolution are less or negligible, v) drug diffusivity is constant, and vi) perfect sink conditions are always attained in the release environment (Parmar and Sharma, 2018).

*In vitro* studies were carried out to test the viability of N2a cells after treatment with the NPs to assess the safety of developed formulations. Both AG and AG NPs did not alter the viability of N2a murine neuroblastoma cells when compared with the untreated control cells.

*In vivo* studies demonstrated that AG NPs administered to TgCRND8 mice were well tolerated, mice had a stable body weight, and no evident side effects were revealed. These data were also in agreement with our previous studies with unloaded human serum albumin NPs, which did not induce an inflammatory response after intracerebral injection, and they are biocompatible and biodegradable (Bergonzi et al., 2016).

The study demonstrated that impaired cognitive functions of TgCRND8 mice in the step-down inhibitory avoidance test and ORT were significantly restored 4 weeks after systemic administration of AG NPs. Because NAF NPs, systemically administered to TgCRND8 mice, penetrated into the brain and NPs were detected in undamaged and damaged brain parenchyma within the amyloid plaques and their surroundings, it might be hypothesized that this functional recovery might be related to protective effects of AG NPs on synaptic plasticity and synaptic proteins, as suggested by Serrano et al. (2014). Furthermore, the immunohistochemical analysis of GFAP-positive astrocytes in the hippocampus of Tg mice evidenced the anti-inflammatory activity of AG when AG NPs were i.p. administered.

In addition, AG ability to prevent or modulate the amyloid aggregation process was evaluated. For this aim, the kinetics of fibril formation were monitored by following the increase of ThT fluorescence emission intensity at 485 nm during the Aβ_1-42_ fibrillation process in the absence or in the presence of different concentrations of AG. AG did not alter significantly the ThT fluorescence spectra from fibrillar Aβ_1-42_ alone. Moreover, the lack of anti-aggregation activity of AG was confirmed by TEM micrographs, evidencing that the proteins after 24 h of aggregation either alone or aggregated in the presence of AG exist as globular micelle-like and prefibrillar assemblies, whereas after 72 h of aggregation, the samples are populated mostly of mature fibrils in both conditions. Finally, AG was unable to dissolve the preformed Aβ fibrils even at very high concentrations. The toxicity of the same protein samples was assessed on N2a cells by using the MTT reduction assay. The amyloid assemblies obtained in the presence of AG or the Aβ preformed fibrils treated with different AG concentrations induced in N2a cells the same toxicity as well as amyloid Aβ. Overall, our study showed that, *in vitro*, the AG molecule, free or loaded into NPs, is unable to interfere with Aβ_1-42_ aggregation process and inhibit the potential toxicity of amyloid aggregates by preventing their binding to the cellular membrane.

## Conclusion

Overall, these data provided strong evidence for the delivery of AG NPs to the brain and their efficacy on cognitive functions. Our results indicate that AG does not show any anti-amyloid activity *in vitro*, and its healthy effects could be the logical outcome of its anti-inflammatory role and reduced astrocyte activation. The neuroinflammation in addition to Aβ deposition is one of the major causes of neuronal loss and synaptic dysfunction in AD; the inflammation can modify the neuron-astrocyte-microglia interaction in CA1 and CA3 (Lana et al., 2017), leading to a decline in hippocampal functions and memory impairment. Considering AD as a multifactorial disease, involving various genetic and environmental risk factors, with resulting amyloid and tau abnormalities, it seems valid to target more than one element in the disease pathology to potentiate the therapeutic outcome. Indeed, a future goal could be to assess the efficacy and safety of combination therapy with the efficient treatment properties of AG added to amyloid- or tau-directed treatments by using NPs as vehicle, following the example of successful treatment combinations for other serious diseases and conditions, such as cancer. In conclusion, our present findings indicate that the developed NPs are transported across the BBB, thus representing valuable and safe vectors for brain delivery systems, and that AG can be released within the brain in a therapeutically relevant concentration. In conclusion, the formulated NPs represent a very attractive strategy because of the extraordinary versatility, nontoxicity, nonimmunogenicity, strong biocompatibility, high biodegradability, astonishing loading capacity of AG, and well tolerability without any serious side effects.

## Data Availability

The raw data supporting the conclusions of this manuscript will be made available by the authors, without undue reservation, to any qualified researcher.

## Ethics Statement

Transgenic (Tg) CRND8 mice harboring a double-mutant gene of APP695 (Chishti et al., 2001) and wild-type (wt) control littermates were used in accordance with the principles of the Basel Declaration and the working document on genetically altered animals—CORRIGENDUM of 24 January 2013—of the National Competent Authorities for the implementation of Directive 2010/63/EU on the protection of animals used for scientific purposes. According to the Italian Regulation (D.Lvo 26/2014), the protocol was revised and approved by the Animal Welfare Body of the University of Florence and licensed by the Italian Competent Authority (Italian Ministry of Health, license number 152/2014-B).

## Author Contributions

ARB, MB, and FC conceptualized the study and funding acquisition. PN, VP, and ML carried out the experiments and their formal analysis. MCB contributed to the analysis of the data. ARB, MB, and FC supervised the data and wrote the manuscript.

## Funding

This work was supported in part by a grant from Ente Cassa di Risparmio di Firenze (Florence, Italy) and Università degli Studi di Firenze. ML was supported by ANCC-COOP/Airalzh ONLUS (Reg. n° 0043966.30-10- 359 2014-u) through University of Florence (D.R.595/2016).

## Conflict of Interest Statement

The authors declare that the research was conducted in the absence of any commercial or financial relationships that could be construed as a potential conflict of interest.
